# Calcium and vitamin-D deficiency marginally impairs fracture healing but aggravates posttraumatic bone loss in osteoporotic mice

**DOI:** 10.1038/s41598-017-07511-2

**Published:** 2017-08-03

**Authors:** Verena Fischer, Melanie Haffner-Luntzer, Katja Prystaz, Annika vom Scheidt, Björn Busse, Thorsten Schinke, Michael Amling, Anita Ignatius

**Affiliations:** 1 Institute of Orthopaedic Research and Biomechanics, University Medical Centre Ulm, Ulm, Germany; 2 Department of Osteology and Biomechanics, University Medical Centre Hamburg-Eppendorf, Hamburg, Germany

## Abstract

Calcium and vitamin-D (Ca/VitD) deficiency is a major risk factor for osteoporosis. It may also contribute to the compromised bone healing frequently observed in osteoporotic patients, since calcium is essential for fracture-callus mineralization. Additionally, clinical data suggest systemic bone loss following fracture, which may aggravate osteoporosis and thus increase the risk for fragility fractures in osteoporotic patients further. However, the role of Ca/VitD in fracture healing and posttraumatic bone turnover has to date been poorly investigated. Here, we studied bone regeneration and posttraumatic bone turnover in C57BL/6 J mice with ovariectomy-induced osteoporosis. Mice were fed a standard or a Ca/VitD-deficient diet. Notably, fracture healing was only marginally disturbed in Ca/VitD-deficient mice. However, deficient mice displayed significantly increased serum parathyroid hormone levels and osteoclast activity, as well as reduced bone mass in the intact skeleton post-fracture, suggesting considerably enhanced calcium mobilization from the intact skeleton during bone regeneration. Ca/VitD supplementation initiated post-fracture prevented posttraumatic bone loss by reducing bone resorption and furthermore improved bone repair. These results imply that adequate Ca/VitD supply post-fracture is essential to provide sufficient calcium for callus-mineralization in order to prevent posttraumatic bone loss and to reduce the risk for secondary fractures in osteoporotic patients with Ca/VitD deficiency.

## Introduction

Osteoporosis is the most common skeletal disorder worldwide and is associated with a progressive decline in bone properties and an increased fracture risk. Calcium and vitamin-D (Ca/VitD) deficiency is a major risk factor for osteoporosis in addition to postmenopausal estrogen decline, old age and immobilization^1^. Vitamin D regulates calcium homeostasis by influencing intestinal calcium absorption, renal calcium reabsorption and bone resorption by osteoclasts^2^. Both vitamin-D deficiency and a low calcium supply result in increased bone resorption to maintain blood calcium, thus reducing bone mass and quality^3,4^. It is estimated that approximately 3 billion people worldwide display vitamin-D deficiency^5^. Due to on-going demographic changes, the number is expected to increase, because in the elderly dietary calcium and vitamin-D intake are mostly insufficient and intestinal calcium absorption and endogenous vitamin-D synthesis are reduced^6–8^. Therefore, to prevent osteoporosis, the National Osteoporosis Foundation recommends Ca/VitD supplementation for individuals at a high risk for osteoporosis, including postmenopausal females aged over 50 with inadequate dietary calcium and vitamin-D intake^9^. However, osteoporosis is frequently undiagnosed until patients have experienced the first fragility fracture and even after fracture, 80–90% of patients do not receive adequate treatment^10,11^. Concluding, even though a sufficient Ca/VitD supply is crucial for skeletal health, undersupply is common, particularly in the osteoporotic elderly^12,13^.

Furthermore, Ca/VitD deficiency may also contribute to fracture-healing complications that are frequently observed in osteoporotic patients^14,15^, because calcium is essential for fracture-callus mineralization^16,17^. Notably, the role of calcium and vitamin D in fracture healing has to date been poorly investigated. There are a limited number of conflicting experimental studies reporting either a negative^18,19^ or no effect^20^ of Ca/VitD deficiency on fracture-callus formation and mechanical callus quality. It is also debated whether Ca/VitD supplementation supports the fracture-healing process^19,21–23^. The lack of research is striking given that 70% of fracture patients display vitamin-D deficiency^24^. Recently, our group investigated fracture healing in a mouse model of hypochlorhydria-induced calcium malabsorption. Notably, bone healing was unaffected, whereas skeletal osteoclast activity was considerably increased post-fracture^23^. These results suggest enhanced posttraumatic bone resorption to mobilize calcium from the intact skeleton when the uptake does not meet the requirements for callus mineralization. In agreement with our results, clinical studies observed systemic bone loss following fracture as indicated by a reduction in bone mineral density (BMD) of up to 15% in the intact skeleton^25,26^. The posttraumatic bone loss may aggravate osteoporosis and explain the significantly increased risk for secondary fractures^27^. We hypothesize that posttraumatic bone loss might particularly occur under Ca/VitD-deficient conditions, however, to the best of our knowledge, related studies are currently lacking^28^.

Therefore, we investigated whether chronic dietary Ca/VitD deficiency compromises bone repair and induces posttraumatic bone loss in a mouse model of ovariectomy (OVX)-induced osteoporosis. We also addressed the question whether dietary Ca/VitD supplementation initiated from the time point of fracture augments fracture healing and prevents posttraumatic bone loss.

## Results

### Effects of dietary calcium and vitamin D on bone turnover in non-fractured ovariectomized mice

**Figure 1:**
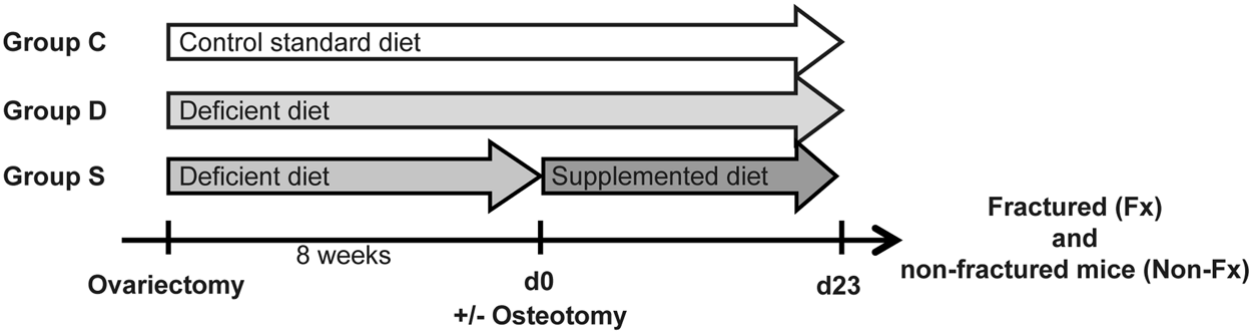
Study design. Following ovariectomy of female C57BL/6 J mice (aged 18 weeks), mice of group C were fed a standard control diet and mice of groups D and S a Ca/VitD-deficient diet. After 8 weeks, mice received a femur osteotomy and group S was transferred to a Ca/VitD-supplemented diet post-fracture. Mice were sacrificed 10 and 23 days after osteotomy. Bone turnover in the intact skeleton of fractured mice (Fx) was compared to that of ovariectomized non-fractured mice (Non-Fx), which received the same feeds.

**Figure 2:**
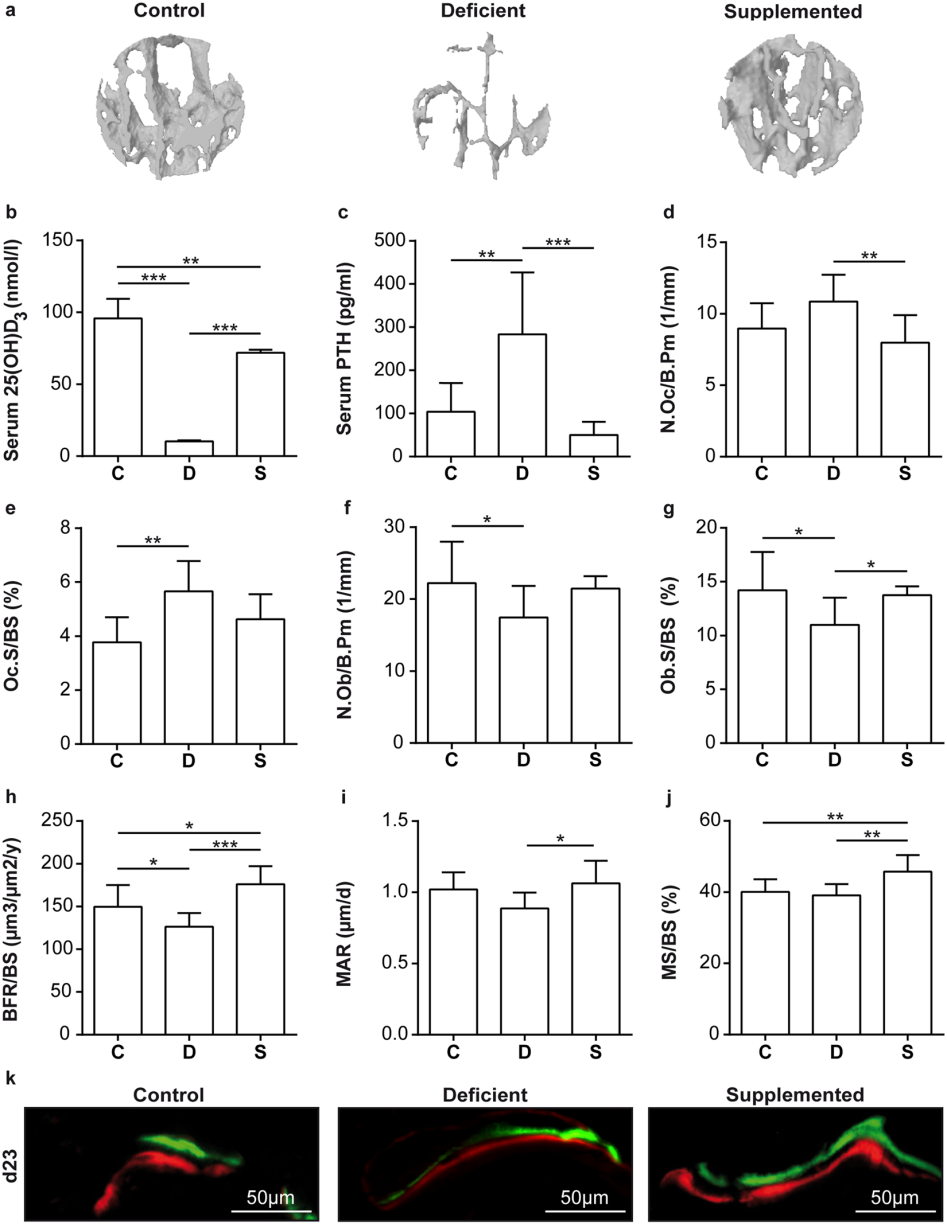
Skeletal examination on day 23 of non-fractured ovariectomized mice fed a control (C), Ca/VitD-deficient (D) or Ca/VitD-supplemented (S) diet. (**a**) Representative 3-dimensional reconstructions (1.3 mm diameter) of trabecular bone in lumbar vertebrae (L2) analyzed by µCT. (**b**) Serum 25(OH)D_3_ (n = 4/group), and (**c**) serum parathyroid hormone (PTH) levels (n = 5–6/group). (**d**) Number of osteoclasts per bone perimeter (N.Oc./B.Pm), (**e**) osteoclast surface per bone surface (Oc.S/BS), (**f**) number of osteoblasts per bone perimeter (N.Ob/B.Pm), (**g**) osteoblast surface per bone surface (Ob.S/BS), (**h**) bone formation rate per bone surface (BFR/BS), (**i**) mineral apposition rate (MAR), and (**j**) mineralized surface per bone surface (MS/BS) examined in lumbar vertebrae (L2; n = 8/group). (**k**) Representative images of fluorescent calcein green and alizarin red labels of lumbar vertebrae (L2). Data presented as the mean ± SD. Significant differences between C, D and S evaluated by ANOVA/Fishers LSD *post-hoc*: *p < 0.05, **p < 0.01, ***p < 0.001.

We first characterized the effects of the feeding protocols on bone turnover in non-fractured ovariectomized mice. We used the same experimental setup as in the subsequent fracture healing experiment but did not induce a fracture (Fig. 1). Briefly, following OVX, the control group C received a standard diet whereas groups D and S received a Ca/VitD-deficient diet. After a further 8 weeks, when bone loss was expected to be manifest and when we planned to induce the fracture, group S was switched to a Ca/VitD-supplemented diet (Fig. 1). After another 23 days (planned fracture-healing period), we performed bone and serum analyses. As expected, bone properties were considerably diminished (Fig. 2a) and serum 25(OH)D_3_ levels were significantly reduced in Ca/VitD-deficient mice compared to mice with standard diet (Fig. 2b). Serum calcium and phosphate levels did not significantly differ between the groups (Supplemental Table S1), whereas serum PTH levels were significantly increased in deficient mice compared to controls (Fig. 2c). As expected, Ca/VitD deficiency decreased bone mass both in the trabecular and cortical compartments (Table 1). µCT analysis of lumbar vertebrae and femurs of deficient mice revealed a significantly reduced cortical thickness, bone volume to tissue volume (BV/TV) and trabecular number (Tb.N), whereas trabecular separation was significantly increased compared to controls (Table 1). In comparison to controls, osteoclast surface in lumbar vertebrae was significantly increased, whereas osteoblast number and surface were significantly reduced in deficient mice (Fig. 2e–g). Dynamic histomorphometry revealed significantly reduced bone formation compared to controls (Fig. 2h,k).

**Table 1:** µCT analysis of lumbar vertebrae and femurs of non-fractured ovariectomized mice.

Parameters		C	D	S
		(n = 8)	(n = 8)	(n = 8)
*Trabecular bone: lumbar vertebrae (L2)*				
Bone mineral density	BMD in HAmg/cm^3^	757 ± 29	722 ± 54	791 ± 45^#^
Bone volume	BV/TV in %	19.4 ± 5.2	12.9 ± 3.6*	19.9 ± 5.8^#^
Trabecular thickness	Tb.Th in mm	0.057 ± 0.007	0.058 ± 0.007	0.068 ± 0.007*^,#^
Trabecular number	Tb.N in 1/mm	3.4 ± 1.0	2.2 ± 0.7*	2.9 ± 0.6
Trabecular separation	Tb.Sp in mm	0.18 ± 0.04	0.23 ± 0.05*	0.23 ± 0.03*
*Cortical bone: femur*				
Bone mineral density	BMD in HAmg/cm^3^	1266 ± 35	1291 ± 55	1331 ± 74*
Cortical thickness	Ct.Th in mm	0.158 ± 0.002	0.154 ± 0.003*	0.160 ± 0.002*^,#^

Data presented as the mean ± SD. C = control diet; D = Ca/VitD-deficient diet; S = Ca/VitD-supplemented diet. ANOVA/Fishers LSD *post-hoc*; *vs. C p < 0.05; ^#^vs. D p < 0.05.

Ca/VitD supplementation abolished the osteo-catabolic effects of the deficient diet (Table 1; Fig. 2a). Compared to deficient mice, serum 25(OH)D_3_ levels were significantly increased, whereas serum PTH levels were significantly reduced in supplemented mice (Fig. 2b,c). However, serum 25(OH)D_3_ levels of supplemented mice were still significantly lower compared to control mice (Fig. 2b). µCT and histomorphometric analyses revealed a significantly increased BMD, BV/TV and trabecular and cortical thicknesses (Table 1) as well as a reduced number of osteoclasts and an increased osteoblast surface in supplemented mice compared to deficient mice (Fig. 2d,g). In relation to these changes, in supplemented mice the BFR/BS and MS/BS were significantly increased compared to both mice with the deficient and control diet, whereas the MAR was only increased compared to deficient mice (Fig. 2h–k). Further µCT and histomorphometric parameters did not differ between non-fractured supplemented and control mice (Table 1; Fig. 2), however, we did not assess the amount of presumed bone loss in the skeleton of the mice prior to supplementation when differences between both groups are expected due to the preceding deficiency.

### Effects of dietary calcium and vitamin D on fracture healing

**Figure 3:**
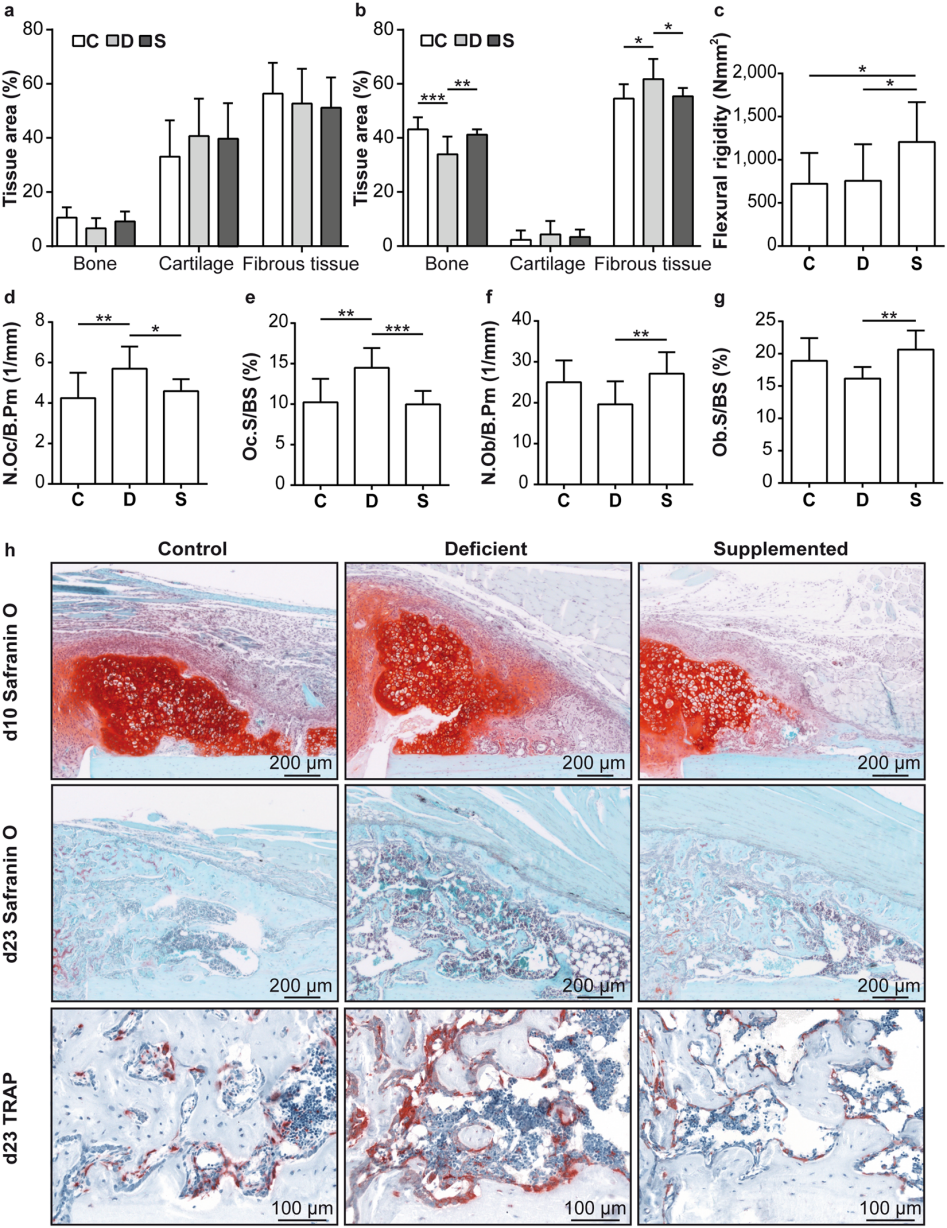
Histomorphometric and biomechanical analysis of fractured femurs in ovariectomized mice fed a control (C), Ca/VitD-deficient (D) or Ca/VitD-supplemented (S) diet. Percentage of bone, cartilage and fibrous tissue in the fracture callus (**a**) on day 10 (n = 6–8/ group) and (**b**) on day 23 (n = 8/group). (**c**) Flexural rigidity of fractured femurs on day 23 (n = 8–10/group). (**d**) Number of osteoclasts per bone perimeter (N.Oc/B.Pm), (**e**) osteoclast surface per bone surface (Oc.S/BS), (**f**) number of osteoblasts per bone perimeter (N.Ob/B.Pm) and (**g**) osteoblast surface per bone surface (Ob.S/BS) in the fracture callus on day 23 (n = 7–9/group). (**h**) Representative images of the fracture callus stained with Safranin O and tartrate-resistant acid phosphatase (TRAP). Data presented as the mean ± SD. Significant differences between C, D and S evaluated by ANOVA/Fishers LSD *post-hoc*: *p < 0.05, **p < 0.01, ***p < 0.001.

We next evaluated the effects of Ca/VitD deficiency on fracture healing in ovariectomized mice. Ten days after osteotomy no significant effects of Ca/VitD deficiency on the callus tissue composition were observed (Fig. 3a,h). However, in the later healing period, Ca/VitD-deficient mice displayed a significantly reduced BMD in the fracture callus compared to control mice (Table 2). BV/TV was not significantly altered (Table 2), whereas in histomorphometry, deficient mice displayed a significantly reduced amount of bone and a significantly increased amount of fibrous tissue in the newly formed callus (Fig. 3b,h). Furthermore, fewer fractures were completely bridged with bone in the deficient group (Table 2). The flexural rigidity of the fractured femurs did not differ significantly between control and deficient mice (Fig. 3c). In the fracture calli, the number and surface of osteoclasts were significantly increased, whereas osteoblast parameters were not significantly affected in the deficient group (Fig. 3d–h). Concluding, these data indicate that in deficient mice the bone content was slightly reduced, but the biomechanical properties of the callus were unaffected, suggesting that bone healing was only moderately disturbed in mice with chronic Ca/VitD deficiency.

**Table 2:** µCT analysis of the fracture callus of ovariectomized mice on day 23 post-fracture.

Fracture callus parameters		C	D	S
		(n = 8)	(n = 9)	(n = 9)
Bone mineral density	BMD in HAmg/cm^3^	371 ± 74	303 ± 48*	343 ± 46
Bone volume	BV/TV in %	20 ± 7	18 ± 5	19 ± 6
Tissue volume	TV in mm^3^	5.7 ± 2.4	7.1 ± 2.7	7.0 ± 1.8
Healed fractures	in % (total healed)	63 (5 of 8)	56 (5 of 9)	77 (7 of 9)

Data presented as the mean ± SD. C = control diet; D = Ca/VitD-deficient diet; S = Ca/VitD-supplemented diet. BMD, BV/TV and TV: ANOVA/Fishers LSD *post-hoc*; Healed fractures: Chi-square test; *vs. C p < 0.05.

**Figure 4:**
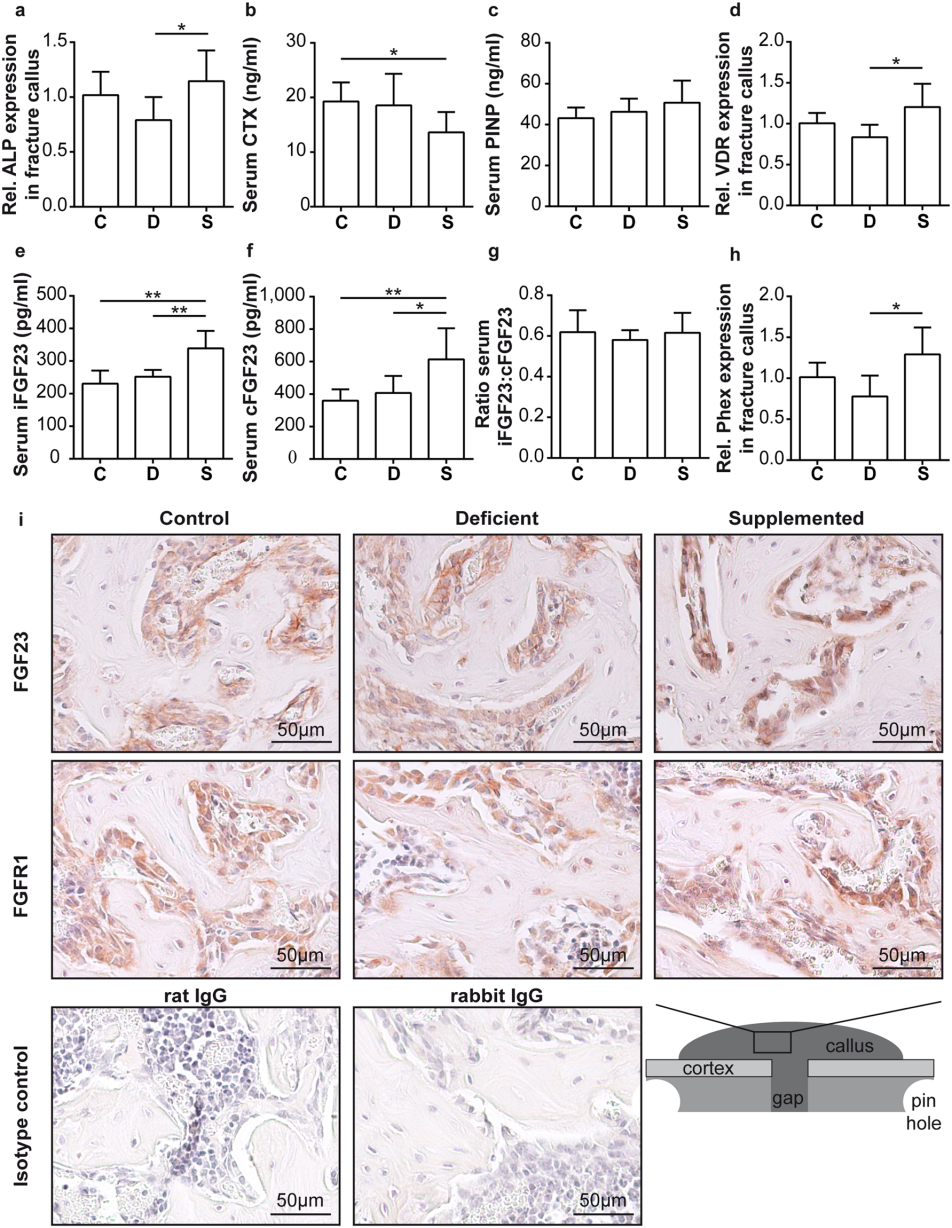
Serum analysis and gene expression and immunohistochemical analyses of the fracture calli of ovariectomized fractured mice fed a control (C), Ca/VitD-deficient (D) or Ca/VitD-supplemented (S) diet on day 23. (**a**) Relative alkaline phosphatase (ALP) gene expression in the fracture calli (n = 4–5/group). (**b**) Serum C-terminal telopeptide of type I collagen (CTX), and (**c**) serum N-terminal propeptide of type I procollagen (PINP) levels (n = 6–7/group). (**d**) Relative vitamin D-receptor (VDR) gene expression in the fracture calli (n = 4–5/group). (**e**) Serum fibroblast intact growth factor 23 (iFGF23) and (**f**) C-terminal FGF23 (cFGF23) levels (n = 5–7/group). (**g**) Ratio of serum iFGF23 to serum cFGF23 levels (n = 5/group). (**h**) Relative phosphate-regulating neutral endopeptidase, X-linked (Phex) gene expression in the fracture calli (n = 4–5/group). (**i**) Representative images of FGF23, fibroblast growth factor receptor 1 (FGFR1) and species-specific isotype control (rat and rabbit IgG) immunostained sections of the fracture calli. For gene expression analyses, β-2-microglobulin was used as the house-keeping gene. Data presented as the mean ± SD. Significant differences between C, D and S evaluated by ANOVA/Fishers LSD *post-hoc*: *p < 0.05, **p < 0.01.

We also investigated whether the negative effects of the Ca/VitD deficiency on bone repair were abolished when Ca/VitD supplement was provided from the time of fracture. Ca/VitD supplementation provoked no significant effects 10 days after fracture (Fig. 3a,h). In contrast, by day 23, Ca/VitD supplementation slightly increased BMD in the fracture calli, but not significantly (Table 2). However, histomorphometric analysis, which, unlike µCT, also captures immature, less mineralized bone, revealed a significantly increased bone fraction and a reduced fibrous tissue fraction in the fracture calli of supplemented mice compared to deficient mice (Fig. 3b,h). Consequently, supplemented mice displayed a significantly increased flexural rigidity (Fig. 3c) and the highest percentage of healed fractures (Table 2), which was not statistically different compared to both other groups since the power of the study was insufficient to detect significant differences with the chi-square test. The higher bone fraction in the callus of supplemented mice resulted from reduced osteoclast and increased osteoblast activities (Fig. 3d–h). This was confirmed by a significantly increased mRNA expression of the osteoblast marker ALP in the fracture callus of supplemented mice compared to deficient mice (Fig. 4a) and by reduced serum levels of the bone-resorption marker CTX compared to control mice (Fig. 4b), however the serum levels of the bone-formation marker PINP were unaffected (Fig. 4c). VDR expression was significantly increased in the callus of supplemented mice compared to deficient mice (Fig. 4d), implying enhanced vitamin-D signalling due to Ca/VitD supplementation post-fracture. Concluding, these results suggest that Ca/VitD supplementation initiated at the time of fracture abolished and even overcompensated the negative effects of chronic Ca/VitD deficiency.

We also determined serum FGF23, a major regulator of phosphate and vitamin-D metabolism^29^. The serum levels of both intact FGF23 (iFGF23) and its inactive C-terminal fragment (cFGF23) were significantly increased in supplemented mice 23 days post-fracture compared to control and deficient mice (Fig. 4e,f), indicating increased systemic FGF23 turnover. However, the ratio of serum iFGF23:cFGF23 levels, representing the biological active FGF23 fraction, did not differ between the groups (Fig. 4g). Confirming this, the protein expression of FGF23 and its main receptor FGFR1 in the fracture callus was not significantly affected by the diet (Fig. 4i). Both FGF23 and FGFR1 were expressed by osteoblasts located on the surface of newly formed bone trabeculae in the callus and by some embedded osteocytes (Fig. 4i). Fracture calli qPCR analysis further confirmed similar FGFR1 and FGF23 mRNA-expression levels in all groups (data not shown). The mRNA expression of Phex, an endopeptidase involved in FGF23 metabolism^30^, was significantly higher in supplemented mice compared to deficient mice (Fig. 4h), corroborating enhanced FGF23 turnover.

### Effects of dietary calcium and vitamin D on posttraumatic bone turnover

**Figure 5:**
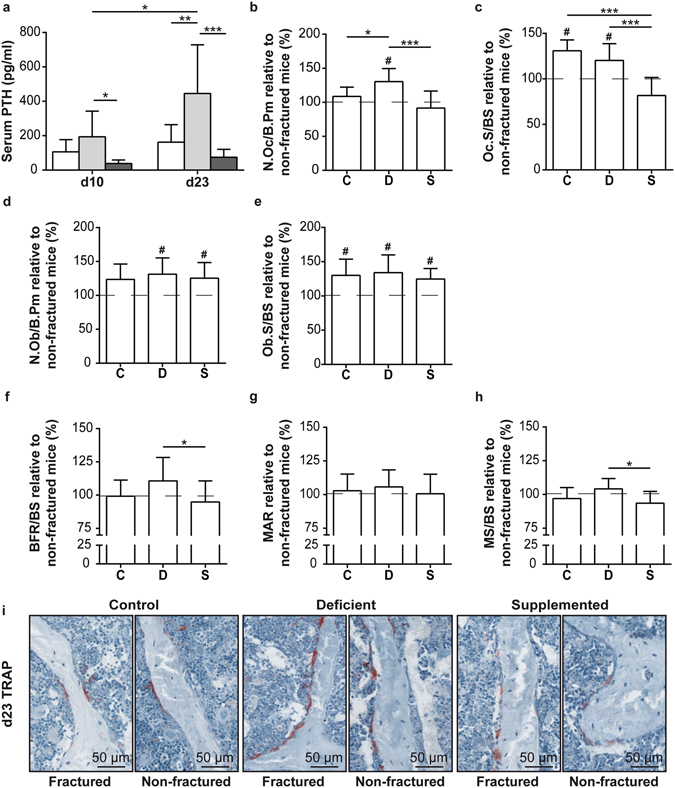
Serum analysis and histomorphometric evaluation of posttraumatic bone turnover in ovariectomized mice fed a control (C), Ca/VitD-deficient (D) or Ca/VitD-supplemented (S) diet. (**a**) Serum parathyroid hormone (PTH) concentrations of fractured mice on day 10 and 23 post-fracture (n = 6–8/group). (**b**) Number of osteoclasts per bone perimeter (N.Oc/B.Pm), (**c**) osteoclast surface per bone surface (Oc.S/BS), (**d**) number of osteoblasts per bone perimeter (N.Ob/B.Pm), (**e**) osteoblast surface per bone surface (Ob.S/BS), (**f**) bone formation rate per bone surface (BFR/BS), (**g**) mineral apposition rate (MAR) and (**h**) mineralized surface per bone surface (MS/BS) of fractured mice relative to non-fractured mice of the same dietary group (dashed line) in percent examined in lumbar vertebrae (L2) on day 23 (n = 7–9/group). (**i**) Representative images of lumbar vertebrae stained with tartrate-resistant acid phosphatase (TRAP) of fractured and non-fractured mice. Data presented as the mean ± SD. Significant differences between C, D and S: *p < 0.05, **p < 0.01, ***p < 0.001 evaluated by ANOVA/Fishers LDS *post-hoc*. ^#^Significantly different (p < 0.05) from non-fractured mice of the same dietary group (dashed line) evaluated by Student’s *t*-test.

We next addressed the question whether dietary calcium and vitamin D influences posttraumatic bone turnover in ovariectomized mice. In deficient mice, PTH serum concentrations continuously increased during fracture healing and were significantly higher compared to the control group C on day 23 (Fig. 5a). In addition, PTH serum levels of fractured mice with deficiency were increased compared to the non-fractured mice (PTH in pg/ml: 446 ± 283 Fx vs. 284 ± 173 Non-Fx) on day 23 (Figs 2c and 5a). In agreement with these data, fractured mice of the deficient-diet group exhibited significantly more osteoclasts in the lumbar vertebrae compared to the fractured control group and to non-fractured mice with Ca/VitD deficiency (dashed line) (Fig. 5b,i). Osteoclast surface was not significantly different between fractured mice with deficient and standard diets, but was significantly increased compared to the corresponding non-fractured mice (dashed line) (Fig. 5c). Osteoblast number and surface were increased in fractured mice of the deficient and supplemented groups compared to non-fractured mice (dashed line) (Fig. 5d,e). Dynamic histomorphometric analysis revealed a relative increased BFR/BS and MS/BS in deficient mice with fracture compared to supplemented mice with fracture (Fig. 5f,h). However, there were no significant differences in dynamic histomorphometric parameters of fractured mice relative to non-fractured mice (dashed line) (Fig. 5f–h).

**Figure 6:**
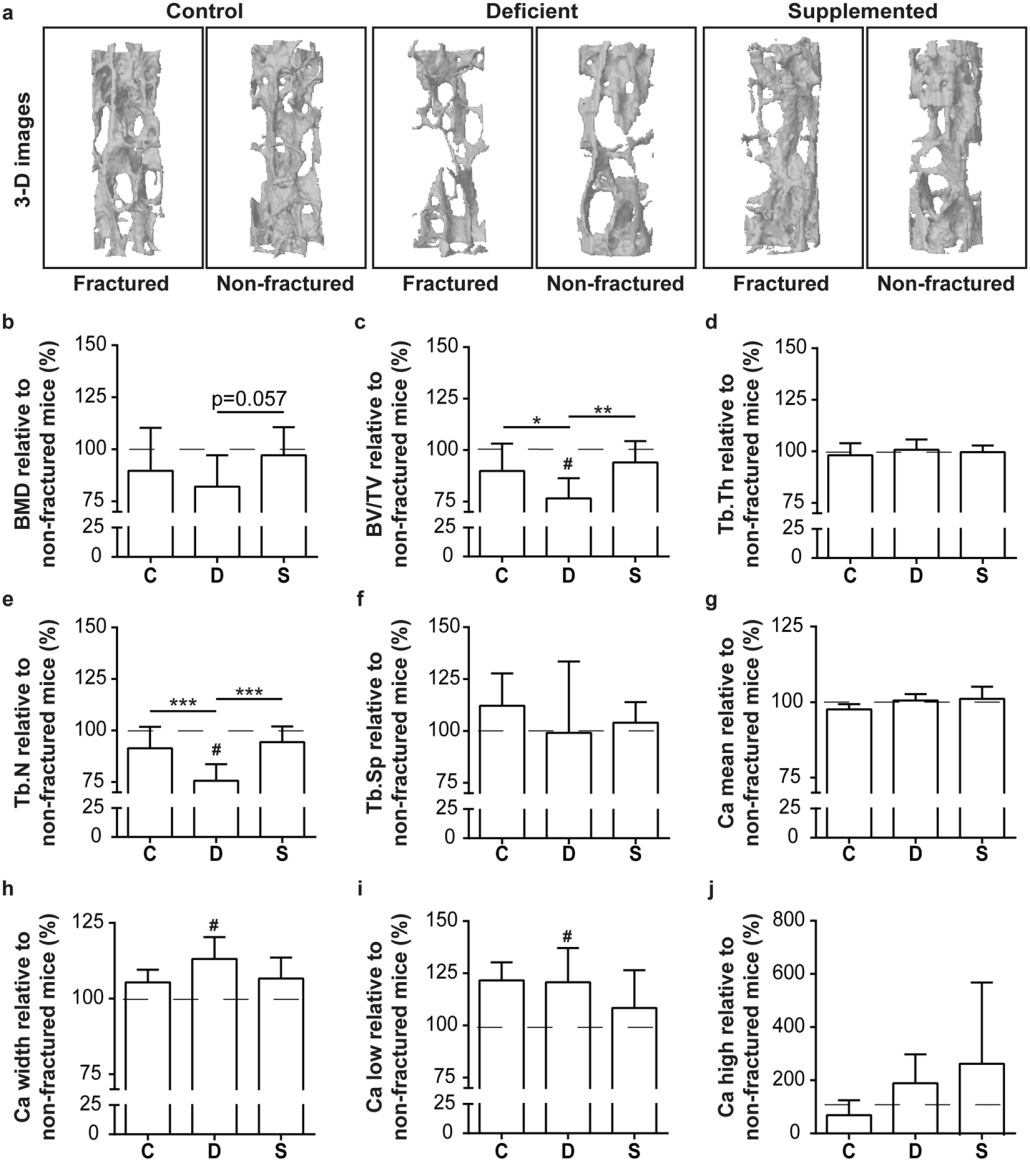
µCT and quantitative backscattered imaging (qBEI) analysis of posttraumatic bone turnover in ovariectomized mice fed a control (C), Ca/VitD-deficient (D) or Ca/VitD-supplemented (S) diet. (**a**) Representative 3-dimensional reconstructions (0.8 mm diameter) of trabecular bone in lumbar vertebrae (L2) analyzed by µCT. (**b**) Bone mineral density (BMD), (**c**) bone volume to tissue volume (BV/TV), (**d**) trabecular thickness (Tb.Th), (**e**) trabecular number (Tb.N), and (**f**) trabecular separation (Tb.Sp) of fractured mice relative to non-fractured mice of the same dietary group (dashed line) in percent examined in lumbar vertebrae (L2) on day 23 (n = 7–9/group). (**g**) Ca mean, (**h**) Ca width, (**i**) Ca low and (**j**) Ca high of fractured mice relative to non-fractured mice of the same dietary group (dashed line) in percent examined in lumbar vertebrae (L2) on day 23 (n = 5/group). Data presented as the mean ± SD. Significant differences between C, D and S: *p < 0.05, **p < 0.01, ***p < 0.001 evaluated by ANOVA/Fishers LDS *post-hoc*. ^#^Significantly different (p < 0.05) from non-fractured mice of the same dietary group (dashed line) evaluated by Student’s *t*-test.

µCT analysis of lumbar vertebrae identified a significantly reduced BV/TV and Tb.N of fractured deficient mice relative to non-fractured mice with deficiency (dashed line) and compared to fractured control mice (Fig. 6a,c,e), thus indicating increased posttraumatic bone loss under Ca/VitD deficient conditions. In addition, qBEI analysis of lumbar vertebrae revealed a significantly increased Ca low and a higher heterogeneity (Ca width) in fractured deficient mice relative to deficient mice without fracture (Fig. 6h,i), suggesting a higher fraction of very low mineralized bone after fracture. Further qBEI parameters did not differ between the dietary groups and between fractured and non-fractured mice (Fig. 6g–j). Taken together, these data suggest a pronounced posttraumatic bone resorption under Ca/VitD deficiency induced by increased PTH serum levels leading to a posttraumatic reduction in bone mass and mineral.

Ca/VitD supplementation abolished the posttraumatic PTH increase (Fig. 5a). In agreement with these findings, the osteoclast activity in the lumbar vertebral bodies of fractured mice with supplementation was significantly reduced compared to fractured mice with the deficient diet (Fig. 5b,c,i). In addition, BMD was increased by trend (p = 0.057) and BV/TV and Tb.N were significantly increased after Ca/VitD supplementation compared to deficient mice (Fig. 6a–c,e). Furthermore, Ca low and Ca width did not differ between fractured supplemented mice and supplemented mice without fracture (Fig. 6h,i). In addition, Ca/VitD supplementation slightly but not significantly reduced Ca width and Ca low, as well as increased Ca high compared to fractured deficient mice (Fig. 6h-j). These data indicate that Ca/VitD supplementation post-fracture ameliorated posttraumatic bone resorption and bone loss.

## Discussion

In this study we investigated the role of calcium and vitamin-D in fracture healing and posttraumatic bone turnover in a mouse model of OVX-induced osteoporosis. Fracture healing was almost not affected in mice with chronic dietary Ca/VitD deficiency. In contrast, these mice did display significantly increased serum PTH levels as well as increased osteoclast activity and reduced bone mass and mineralization in the intact skeleton post-fracture, indicating increased calcium mobilization from the intact skeleton during bone healing. Ca/VitD supplementation initiated at time of fracture improved healing and abolished posttraumatic bone resorption. Therefore, our study demonstrates the importance of a sufficient calcium and vitamin-D supply during fracture healing to provide sufficient dietary calcium for callus mineralization and to prevent posttraumatic bone loss, and is, thus, clinically highly relevant.

To mimic chronic Ca/VitD deficiency in postmenopausal females, we used ovariectomized mice that received a diet without Ca/VitD. In agreement with the limited earlier studies in rodents^31–33^, we demonstrated that in non-fractured mice Ca/VitD deficiency significantly increased systemic PTH levels and bone resorption and decreased bone formation, resulting in a reduced cortical and trabecular bone mass. Ca/VitD supplementation for 3 weeks almost completely abolished the catabolic effects of the 8 weeks of deficiency, in agreement with experimental studies in rats and mice^34–37^. Additionally, several clinical trials of Ca/VitD supplementation in aged, postmenopausal females described increased BMD and a reduced fracture risk^38,39^. Therefore, our mouse model mimics clinical Ca/VitD deficiency in postmenopausal females, thus making it suitable to study fracture healing and posttraumatic bone turnover under these conditions.

Because calcium is essential for callus mineralization during bone regeneration^16,17^, we investigated whether Ca/VitD deficiency influenced bone repair. The Ca/VitD-deficient mice displayed moderately impaired fracture healing as indicated by a reduced bone fraction and increased osteoclast numbers in the fracture callus. The high systemic PTH levels in deficient mice may be responsible for the significantly increased osteoclast activity in the callus. Regarding the effects of Ca/VitD deficiency on bone healing, there are only a limited number of rat studies, some published much earlier, which reported contradictory effects on callus mineralization and biomechanical bone properties^18–20,40^. In agreement with our results, patients with impaired healing and non-unions frequently displayed lower vitamin-D serum levels compared to patients with uneventful healing^41–43^. Importantly, we demonstrated that Ca/VitD supplementation abolished the negative effects of the previously deficient diet, as demonstrated by an increased fracture-callus bone fraction. Supporting this, osteoblast numbers and ALP in the callus were increased, whereas osteoclast numbers and serum CTX were diminished, due to reduced serum PTH levels in supplemented mice. Furthermore, the bending stiffness of the healed femurs was considerably increased due to a higher percentage of bony bridged fracture gaps, further confirming improved fracture healing following Ca/VitD supplementation. Again, experimental studies in rodents reported contradictory results regarding the effects of Ca/VitD administration on fracture healing^21–23^, which may result from the widely varying experimental conditions. However, our results are in agreement with clinical data. In a prospective, randomized study, postmenopausal females who received Ca/VitD supplementation displayed higher BMD in the fracture region after 6 weeks compared to patients receiving placebo^44^.

It is hypothesized that vitamin D exerts its positive effects on bone regeneration mainly indirectly through its endocrine action on calcium homeostasis^2^, thus increasing the calcium supply for fracture callus mineralization. However, direct vitamin-D effects on bone are also widely discussed, because osteoblasts express the VDR and its expression on these cells is directly regulated by biologically active 1,25(OH)_2_D_3_^45,46^. Several *in-vitro* studies demonstrated that 1,25(OH)_2_D_3_ binding to the VDR enhanced osteoblast differentiation and mineralization, which is often indicated by the increased activity of the osteoblast differentiation marker ALP^47,48^. The present study demonstrated increased expression of VDR and ALP in the fracture callus of supplemented mice. Based on these results, we propose that enhanced 1,25(OH)_2_D_3_/VDR signalling locally in the fracture callus may also contribute to Ca/VitD-treatment induced fracture-healing improvement. However, further studies need to investigate the exact mechanisms responsible for the changes resulting from the dietary treatments.

We also investigated FGF23 because it is a regulator of mineral and vitamin D metabolism and was proposed to be important for efficient bone healing^49^. In supplemented mice, both intact FGF23 (iFGF23) and the inactive proteolytic C-terminal fragment (cFGF23) serum levels were significantly increased, indicating enhanced FGF23 turnover. However, the iFGF23:cFGF23 ratio was unaltered, possibly indicating unchanged biologically active FGF23. Supporting this, FGF23 and FGFR1 expression in the fracture callus was not influenced by supplementation, suggesting that local FGF23 signalling was unaffected. The increased endopeptidase Phex mRNA expression in the fracture callus, which is thought to be involved in FGF23 cleavage^30^, supports enhanced FGF23 turnover in supplemented mice. However, further investigations are needed to clarify these observations.

We recently demonstrated that while fracture healing was unaffected in mice with intestinal calcium malabsorption, osteoclast activity in the intact skeleton was significantly increased, suggesting increased posttraumatic bone resorption^23^. In the present study, we confirmed these results. Ca/VitD-deficient mice similarly exhibited increased PTH serum levels post-fracture and enhanced osteoclast activity in the intact skeleton compared to non-fractured mice. In addition, PTH serum levels were higher in fractured mice in comparison to non-fractured mice with deficiency, thus corroborating fracture-induced changes in PTH serum levels. In agreement with our findings, increased post-fracture PTH serum levels were observed in several clinical studies^50–52^. Notably, elevated serum PTH could still be detected a year post-fracture in postmenopausal females^52^. Several clinical studies observed systemic bone loss after fracture^25,26,53^, thus possibly contributing to the considerably increased risk for secondary fractures^27,54^. One such study demonstrated that reduced bone mass after fracture significantly correlated with a reduced dietary calcium intake as well as with low vitamin-D serum levels^53^, indicating that Ca/VitD deficiency may aggravate posttraumatic bone loss. In agreement with these clinical findings and our previous data, we similarly detected altered bone mineralization in the intact skeleton post-fracture. Deficient mice with fracture exhibited a reduced bone mass and a higher percentage of less mineralized bone areas compared to non-fractured mice with deficiency, thus indicating posttraumatic bone loss. This suggests that Ca/VitD-deficient mice release calcium from their intact skeleton to provide sufficient calcium for callus mineralization. The increased posttraumatic calcium mobilization may explain why healing was only marginally compromised by Ca/VitD deficiency, however, at the clear expense of the intact skeleton. Ca/VitD supplementation after fracture prevented posttraumatic bone loss by reducing serum PTH levels and osteoclastic bone resorption. Supporting this, a clinical study reported decreased PTH serum levels in elderly patients treated post-fracture with Ca/VitD, whereas PTH levels were elevated in the placebo group^51^. These findings and our results corroborate the clinical therapeutic requirement for Ca/VitD supplementation post-fracture.

In conclusion, our results indicate that under Ca/VitD deficiency, calcium is increasingly mobilized from the intact skeleton to allow efficient fracture healing. The resulting posttraumatic bone loss may contribute to the 3-fold increased risk of secondary fractures in osteoporotic patients, which are associated with a dramatic increase in patient morbidity and mortality and a reduction in the quality of life^55^. Ca/VitD supplementation initiated at the time of fracture augmented bone repair and prevented posttraumatic bone loss, demonstrating the clinical need for Ca/VitD treatment to reduce fracture-healing complications in osteoporotic patients and to prevent fracture-induced bone loss.

## Materials and Methods

### Study design

The animal experiment was approved by the Local Ethical Committee (No. 1184, Regierungspräsidium Tübingen, Germany) and was in compliance with international recommendations for the care and use of laboratory animals (ARRIVE guidelines and EU Directive 2010/63/EU for animal experiments). Female C57BL/6 J mice were purchased from JanvierLabs (Saint-Berthevi, France). Mice were given *ad libitum* access to water and food, receiving a standard mouse feed (R/M-H, V1535–300, Ssniff Spezialdiäten, Soest, Germany). When aged 16 weeks, the feed was switched to a phytoestrogen-reduced diet (R/M-H, V1554-300, Ssniff). OVX was performed when aged 18 weeks, and the mice were randomly assigned to three groups (Fig. 1). The control group C was continuously fed with the phytoestrogen-reduced diet after OVX, whereas groups D and S received a Ca/VitD-deficient diet (S8276-E710, 0.25% calcium, 0 IU/kg vitamin D, Ssniff) in order to study the proof-of-principle of a severe Ca/VitD deficiency on bone repair and posttraumatic bone turnover, although a milder insufficiency would be more reflective of the majority of osteoporotic patients. All mice received a femur osteotomy 8 weeks after OVX. Group S was transferred to a Ca/VitD-supplemented diet (S8276-E712, 2.0% calcium, 2000 IU/kg vitamin D, Ssniff) immediately after surgery. We included additional control groups, which were treated as described above but received no osteotomy (non-fractured mice; Fig. 1). Mice were euthanized on days 10 and 23 (n = 5–10), and fracture healing and posttraumatic bone turnover were evaluated.

### Surgery

OVX and femur osteotomy were performed under general anesthesia using 2% isoflurane (Florene, Abbott, Wiesbaden, Germany). For analgesia, mice received 25 mg/ml tramadol hydrochloride (Tramal®, Gruenenthal, Aachen, Germany) in the drinking water, starting one day preoperatively until three days postoperatively. Before surgery, mice received one subcutaneous injection of the antibiotics clindamycin-2-dihydrogenphosphate (45 mg/kg, Ratiopharm, Ulm, Germany). Mice were bilaterally ovariectomized by ligation of the oviduct and removal of the ovary. Femur osteotomy was performed as described previously^23^. Briefly, at the midshaft of the right femur an osteotomy gap (0.4 mm) was created and stabilized using an external fixator (axial stiffness 3 N/mm, RISystems, Davos, Switzerland). The mice were injected after 10 days with calcein green (0.03 g/ml, Sigma-Aldrich, St. Louis, USA) and after 15 days with alizarin red (0.045 g/ml, Sigma-Aldrich) for dynamic bone histomorphometry.

### Serum analysis

Blood samples were obtained on the day of euthanasia by heart puncture. Serum 25-hydroxy vitamin D_3_ (25(OH)D_3_), the bone-resorption marker C-terminal telopeptide of type I collagen (CTX) and the bone-formation marker N-terminal propeptide of type I procollagen (PINP) were determined using commercially available enzyme-immunoassay (EIA) kits (25-hydroxy vitamin D EIA, AC57F1; RatLaps^TM^ (CTX-I) EIA, AC-06F1; Rat/Mouse PINP EIA, AC-33F1; all Immunodiognostic Systems, Frankfurt, Germany). Parathyroid hormone (PTH) and fibroblast growth factor 23 (FGF23; intact and C-terminal) serum levels were determined using enzyme-linked immunosorbent assay (ELISA) kits (Mouse PTH 1-84 ELISA Kit 60-2305; Mouse/Rat FGF-23 (C-Term) ELISA Kit 60-6300; Mouse/Rat FGF-23 (Intact) ELISA Kit 60-6800; all Immutopics Inc., San Clemente, USA). Colorimetric kits were used to determine serum calcium and phosphate levels (QuantiChrom^TM^ Calcium Assay Kit, DICA-500; QuantiChrom^TM^ Phosphate Assay Kit, DIPA-500, both BioAssay, Hayward, USA).

### Biomechanical testing

To evaluate biomechanical competence, femurs explanted at day 23 were tested by a non-destructive, three-point-bending test using a material-testing machine (1454, Zwick, Ulm, Germany)^23^. Briefly, after fixator removal, an axial load with a maximum load of 4-N was applied at the midshaft of the femurs and the load and deflection were recorded. Flexural rigidity of the bones was calculated using the linear elastic part of the load-deflection curve.

### Micro-computed tomography (µCT) analysis

On day 23, femurs and lumbar vertebrae were imaged using a µCT scanning device (Skyscan 1172, Kontich, Belgium) operating at a resolution of 8 µm and a voltage of 50 kV and 200 µA. Calibration (hydroxyapatite (HA) phantoms: 250 and 750 mg/cm^3^) and global thresholding (callus and cortical bone: 641.9 mg HA/cm^3^; trabecular bone: 394.8 mg HA/cm^3^) were conducted and intact femurs, lumbar vertebrae and the fracture calli were evaluated as described previously^56^. Common standard parameters of the American Society for Bone and Mineral Research (ASBMR) were determined using Skyscan software (NRecon, DataViewer, CTAn)^57^. To evaluate bony bridging of the fracture gap, the number of bridged cortices per callus was evaluated in two peripendicular planes using µCT images. When ≥3 bridged cortices per callus were observed, the fracture was considered as ‘successfully healed’.

### Histomorphometry and immunohistochemistry

Femurs and lumbar vertebrae explanted on days 10 and 23 were embedded in methyl methacrylate or paraffin^56^. For histomorphometric analysis of the fracture callus, the specimens were stained using Safranin O and the relative amounts of bone, cartilage and fibrous tissue in the whole callus between the two inner pinholes were determined using image-analysis software (Leica MMAF 1.4.0 Imaging System, Leica, Wetzlar, Germany). Cellular parameters were evaluated in femurs and lumbar vertebrae according to the ASBMR guidelines at day 23 after staining with tartrate-resistant acid phosphatase or toluidine blue^58^. Osteoblasts and osteoclasts were counted in a 1.8 × 0.9-mm region in the middle of the fracture callus and a 0.6 × 0.6-mm region centered in the second lumbar vertebral body using the image analysis software Osteomeasure® (OsteoMetrics, Decatur, USA). The mineral-apposition rate (MAR), bone-formation rate per bone surface (BFR/BS) and mineralized surface per bone surface (MS/BS) were determined in the second lumbar vertebral body (d23; 0.6 × 0.6 mm centered region) using Osteomeasure® image analysis (OsteoMetrics).

Paraffin-embedded sections of fractured femurs (d23) were immunohistochemically stained for FGF23 and fibroblast growth factor receptor 1 (FGFR1) using the following antibodies and dilutions: rat anti-mouse FGF23, 1:100 (MAB26291, R&D systems Inc., Minneapolis, USA), rabbit anti-mouse FGFR1/CD331, 1:50 (PA5-25979, Invitrogen, Thermo Fisher Scientific, Waltham, USA), goat anti-rabbit immunoglobulin G (IgG) (H + L) biotin-conjugated, 1:100 (B2770, Invitrogen), and goat anti-rat IgG (H + L) biotin-conjugated, 1:100 (A10517, Invitrogen). Species-specific IgGs were used for isotype controls. Signal amplification was performed using the avidin-biotin complex (Vector, Burlingame, USA). Substrate was detected using NovaRed (Vector) and the sections were counterstained using hematoxylin (Waldeck, Münster, Germany).

### Quantitative backscattered electron imaging (qBEI) analysis

To determine mineral content and distribution in lumbar vertebrae, qBEI was performed as described previously^23,59^. Briefly, segments of lumbar vertebrae embedded in methyl methacrylate (d23) were polished, coated with carbon and analyzed using a scanning electron microscope (LEO 435 VP, LEO Electron Microscopy Ltd., Cambridge, England) operating at 20 kV and 665 pA at a constant working distance (BSE Detector, Type 202, K.E. Developments Ltd., Cambridge, England) with a 2.29-µm pixel size. For calibration, an aluminium carbon standard was used^60^. From the generated grey value images we calculated the mean calcium content (Ca mean wt%), the heterogeneity of mineralization (Ca width wt%), the fraction of highly (Ca high %) and poorly (Ca low %) mineralized bone areas of the lumbar vertebrae.

### Gene-expression analysis of the fracture callus

On day 23, the fracture calli were harvested and stored in RNAlater^TM^ (Sigma-Aldrich) at 4 °C. Following RNAlater^TM^ removal, the fracture callus was snap-frozen in liquid nitrogen and pulverized in a vibration mill at 30 Hz for 1.5 minutes. Total RNA was isolated using the TRIzol Plus RNA Purification Kit (Ambion, Thermo Fisher Scientific, Waltham, USA) according to the manufacturer’s instructions plus an additional DNA-digestion step using the RNase-Free DNase Set (50) (Qiagen, Hilden, Germany). In total, 1 µg of isolated RNA was transcribed into cDNA using the Omniscript RT kit (Qiagen). Quantitative polymerase chain reaction (qPCR) was performed using the Brilliant Sybr Green qPCR Master Mix Kit (Stratagene, Amsterdam, Netherlands) according to the manufacturer’s protocol. Alkaline phosphatase (ALP, F: 5′-GCTGATCATTCCCACGTTTT-3′ and R: 5′-GAGCCAGACCAAAGATGGAG-3′), vitamin D-receptor (VDR, F: 5′-GGGCTTCCACTTCAACGCTA-3′ and R: 5′-CATGCTCCGCCTGAAGAAAC-3′), phosphate-regulating neutral endopeptidase, X-linked (Phex, F: 5′-TTCCCCAGTTTGACTGGCTG-3′ and R: 5′-TCTCCGAGGGACCAATGTCT-3′), FGF23 (F: 5′-ACAGGAGCCATGACTCGAAG-3′ and R: 5′-GCAATTCTCTGGGCTGAAGT-3′), and FGFR1 (F: 5′-TGACGACGACGATGACTCCT-3′ and R: 5′-AGCTACAGGCCTACGGTTTG-3′) expression were normalized to the housekeeping gene β-2-microglobulin (F: 5′-ATACGCCTGCAGAGTTAAGCA-3′ and R: 5′-TCACATGTCTCGATCCCAGT-3′). The normalized relative amounts of targets were then compared to the target signals of the control group using the delta-delta CT method with PCR-efficiency correction using LinRegPCR software (Heart Failure Research Center, Academic Medical Center, Amsterdam, Netherlands) as described previously^61^.

### Statistical Analysis

Results are presented as the mean ± standard deviation (SD). Statistical analysis was performed using GraphPad Prism 6 software (GraphPad Software, Inc., La Jolla, USA). Normal distribution of data was tested by Shapiro-Wilk normality test. Data were analysed for significance either by one-way analysis of variance (ANOVA) and Fishers LSD *post-hoc* when three groups were compared to each other or by Student’s *t*-test when two groups were compared. For the comparison of fractured and non-fracture mice, the values of the fractured mice were indicated relative to the values of the non-fractured mice of the same dietary group in percent (%). Data of healed fractures were analysed by chi-square test. The level of significance was set p < 0.05. The number of samples for each experiment is indicated in the figure legends and tables. Sample size was calculated based on the main outcome parameter flexural rigidity of the fractured femur (power: 80%, alpha = 0.05) obtained from previous studies^23^.

### Data availability statement

All relevant data are included within the manuscript. Data can be provided upon request.

## Electronic supplementary material

Supplementary information
