# Publisher Correction: Calcium and vitamin-D deficiency marginally impairs fracture healing but aggravates posttraumatic bone loss in osteoporotic mice

**DOI:** 10.1038/s41598-018-35539-5

**Published:** 2018-11-16

**Authors:** Verena Fischer, Melanie Haffner-Luntzer, Katja Prystaz, Annika vom Scheidt, Björn Busse, Thorsten Schinke, Michael Amling, Anita Ignatius

**Affiliations:** 1 Institute of Orthopaedic Research and Biomechanics, University Medical Centre Ulm, Ulm, Germany; 2 Department of Osteology and Biomechanics, University Medical Centre Hamburg-Eppendorf, Hamburg, Germany

Correction to: *Scientific Reports* 10.1038/s41598-017-07511-2, published online 03 August 2017

In the original version of this Article, the author Annika vom Scheidt was incorrectly indexed. This error has now been corrected.

