# Supplementary material for: Calcium and vitamin-D deficiency marginally impairs fracture healing but aggravates posttraumatic bone loss in osteoporotic mice
Source: Sci Rep. 2017 Aug 3;7:7223. doi: 10.1038/s41598-017-07511-2 (PMC5543125; doi:10.1038/s41598-017-07511-2)
Supplement: Supplementary file 1 — Supplementary information [file 41598_2017_7511_MOESM1_ESM.pdf]

Calcium and vitamin-D deficiency marginally impairs fracture healing but aggravates posttraumatic bone loss in osteoporotic mice

Verena Fischer (M.Sc.)[1], Melanie Haffner-Luntzer (PhD)[1], Katja Prystaz (M.Sc.)[1], Annika vom Scheidt (M.Sc.)[2], Björn Busse (PhD)[2], Thorsten Schinke (Professor, PhD)[2], Michael Amling (Professor, MD)[2], Anita Ignatius (Professor, DVM)[1,*]

[1]Institute of Orthopaedic Research and Biomechanics, University Medical Centre Ulm, Ulm, Germany

[2]Department of Osteology and Biomechanics, University Medical Centre Hamburg-Eppendorf, Hamburg, Germany

**Blood serum parameters**		**C** (n=5-6)	**D** (n=6)	**S** (n=6)
Calcium	Ca in mg/dl	11.3 ±1.1	10.7 ±0.7	11.5 ±0.3
Phosphate	PO in mg/dl	18.0 ±1.6	18.4 ±1.2	17.3 ±1.4

Supplemental Table S1. Blood serum analysis of non-fractured ovariectomized mice. Data presented as the mean ± SD. C = control diet; D = Ca/VitD-deficient diet; S = Ca/VitD-supplemented diet. ANOVA/Fishers LSD *post-hoc.*
